# Historical Account of the Progress of Chemistry, as Applicable to Medicine; from June 1818 to January 1819

**Published:** 1819-02

**Authors:** 


THE LONDON

### Medical and Physical Journal.

2 OF VOL. XLI.] FEBRUARY, 1819. [NO. 240.

" For many fortunate discoveries in medicine, and for the detection of nume-
" rous errors, the world is indebted to the rapid circulation of Monthly
" Journals; and there never existed any work to which the Faculty in
" EUROPE and AMERICA were under deeper obligations than to the
" Medical and Physical Journal of London, now forming a long, but an
" invaluable, series."—RUSH.

### *Historical Account of the Progress of Chemistry, as applicable to Medicine; from June* 1818 *to January* 1819.

" All material substances are either combinations, or the particles of which combinations are formed; these particles, termed atoms, are indivisible and immutable: were it not thus, all the objects we contemplate would long since have been destroyed.—We must not attribute to these atoms any of the qualities of those bodies which are evident to our senses, except density, magnitude, figure, and what necessarily appertains to figure: for all the qualities of sensible bodies change, but those of atoms are unalterable.—It is from the combination of atoms possessed of different qualities, in different numbers and relative proportions, that all the objects we perceive are formed; just as the varieties of expression in written language are composed of the letters of the alphabet variously arranged."

EPICURUS, apud *DIOGENES LAERTIUS, (passim).*

NO reflections lead to more exalted ideas of the power of the human mind, than those excited by contemplation of the opinions which philosophers in different ages have formed respecting the nature of physical objects, and the laws by which their actions are regulated, solely from observation of their evident qualities under natural circumstances: opinions, the establishing of the truth of which has conferred honour on subsequent votaries of science, who have been enabled to effect this only after long and laborious investigation, aided by all the additional powers which the arts can supply to the natural faculties of man. Many erroneous notions have certainly been adopted from such a mode of contemplating nature; but, on the contrary, many of the most profound and important truths have thus been developed. The consideration of the opinions of Epicurus respecting the general nature of the material universe will evince the correctness of this observation. The mutual and relative influence of preconception and experiment in the acquisition of knowledge, constitutes an interesting subject for philosophical enquiry, and much benefit may be anticipated from its pursuit: we had collected some observations tending to show its importance, but we find the relation of them would occupy more space than could with propriety be consigned to a subject not immediately connected with the practice of medicine. The hint may be useful to some of our readers. Further reflection has led us to consider that the science of chemistry is too extensive in its application, and too rapid in its progress at the present period, to permit a sufficient account of it to be comprised in a Journal properly devoted to medical knowledge: we have, therefore, determined to treat of it, in future, merely as it immediately refers to physiology and the practice of medicine.

There is, however, some difficulty in determining in what manner, and to what extent, chemistry is applicable to medicine. There have been physicians of eminent talents, in all parts of Europe, at various periods since the commencement of the fifteenth century, who have endeavoured to explain the phenomena of health and disease on principles deduced from that science, and to correct the latter by chemical agents alone; whilst others have excluded from medical reasonings all principles which are not founded on the ideas conveyed by the terms sensibility, excitability, and vital actions. A general account of the origin and progress of these opinions may not be devoid of interest and utility.

When science began to be cultivated in Europe in the middle ages, the attention of its votaries was chiefly directed to the investigation of the composition and qualities of natural bodies. The Arabians, who were at that period the only men who studied natural philosophy in this part of the world, had long cultivated chemistry; and many chemical remedies were employed by the physicians of that nation: but it appears from the works of GEBER,* the most intelligent of the chemists of the school of Bagdat with whose writings we are acquainted, that their efforts were principally directed to the discovery of the philosopher's stone and the elixir of life. Such also were the views which directed the labours of ARNALD of Villa-Nova, and his pupil RAYMOND LULLY of Majorca, who were the first Europeans that devoted themselves to the study of this science. It is easy to imagine how readily men who were

* There are several chemical tracts in the library of the British Museum which are attributed to Geber.

able to reduce the metallic oxides, and thus form brilliant metals from gross earths, would, in the early state of such a science, be led to suppose that the more precious metals might be formed by similar operations. The desire of fame might also induce them to impose on others a belief that they actually possessed this power. Thus, Arnald is said to have converted iron into gold, at Rome; and Lully to have effected a similar operation before Edward the First, in London. The alchemists of that period were chiefly the *Rosicrucians,* whose wild and vivid imaginations, heated by the fables of the East, in an age expressly calculated to favour their influence, led them to form the most extravagant notions respecting the nature of physical objects and the powers of the human mind. The idea of being able to discover a substance, (with the assistance of gnomes, sylphs, and other visionary spiritual beings, whom they supposed to govern the elements, and over whom they believed man might exercise dominion,) the use of which would render the human body insusceptible of disease and dissolution, was an effort of imagination but little beyond that which embraced the idea of the philosopher's stone. The more rational manner in which this science was studied, at a somewhat later period, by Roger Bacon of Oxford, and Albert of Cologne, had but little influence in checking the progress of those fascinating delusions. But little benefit was, therefore, derived from the study of chemistry beyond that described by Lord Bacon, when he compared the alchemists to those husbandmen, who, in searching for a treasure supposed to be hidden in their land, by turning up and pulverising the soil, rendered it fertile,—until the early part of the fifteenth century, when several chemical medicines of utility were described by Basil Valentine, the author of the celebrated *Triumph-wagen der Antimonii.* Even Cornelius Agrippa professed magic, and endeavoured to combine alchemy with metaphysical philosophy. Paracelsus was the first who endeavoured to explain the nature of the composition of the human body and its functions, solely from chemical principles; and to deduce from this science specifics for all the varieties of disease. He was guided in these views by his hypothesis, that salt, metals, and sulphur, formed the elements of the human body; sol, or gold, he considered to predominate in the heart; luna, or silver, in the brain; mercury, in the lungs; saturn, or lead, in the spleen; and that the alkaëst, or universal solvent, reigned in the liver. All these agents were supposed to be under the dominion of an *Archeus,* which resided in the stomach, and performed the part of an alchemist. The notion he held respecting the manner in which calculous concretions are formed in the urinary passages is, however, by no means contemptible: he compared it to the deposition of tartar from wine. Several physicians rose up to vindicate the doctrines of Hippocrates and Galen, and to banish from practice the use of the remedies derived from the new science; the principal of whom were ERASTUS, DE VINCENCIUS, LINACRE, and ZWINGER;* but, influenced either by the extraordinary and beneficial effects of chemical remedies in some desperate cases, or by the force of popular opinion, they at length were themselves disposed to have recourse to their use.

A new sect now appeared, whose doctrines were a combination of the mysteries of the cabalistic art with the principles of alchemy; amongst whom the most eminent were DIGBY, KELLY, and DEE, in England; and DU CHESNE,† CASTELLUS,‡ MAYERNE, Oswald CROLLIUS, MYNSICHT,§ and SCHROEDER,|| on the continent of Europe.

Several physicians of considerable talents were disposed to accommodate the use of chemical remedies with the doctrines of the ancients; the principal of whom were Angelus SALA,¶ of whom it was said, *Primus chemicorum desiit ineptire;* SENNERTUS,** TACHENIUS,†† HARTMANN,‡‡ who also delivered lectures on the application of chemistry to medicine; as did ROLFINCK at Jena, and RIVIERE at Montpellier. GUIBERT, GASSENDI, and KEPLER, contributed also much to the rescue of medicine from the dominion of alchemy. But the most powerful reformer of this class was Van HELMONT: instead of explaining all the vital phenomena on the principles of mineral chemistry, he sought for illustrations of them in the operations of the vegetable world. Thus, digestion and secretion were compared to fermentation, which, as well as the other operations of the human body, were governed by an *Archeus* situated in the stomach, to which qualities somewhat similar to those since assigned

---

* *Physiologia Medica;* Basil, 1610.

† *Pharmacopœia Dogmaticorum Restituta;* Paris, 1607.

‡ *Calchantion Dodecaperion;* Romæ, 1619.

§ *Thesaurus et Armamentarium Medico-chymicum;* Hamburg, 1631.

|| *Pharmacopœia Medico-physica;* Ulm, 1641.

¶ *Anatomia Antimonii, id est, dissectio tam dogmatica quam hermetica antimonii, usum, proprietates, et vires ejus declarans;* Lugd. Batav. 1617.

** *De Concensu ac Dissensu Chymicorum cum Galeno et Aristotele;* in Opera omn. Lugdun. 1650.

†† *Hippocrates Chymicus;* Venetæ, 1666.

‡‡ *Praxis Chymiatrica;* Francofurti, 1690.

to another imaginary existence—the *vital principle*, or to the *vis medicatrix naturæ* of STAHL, were attributed. The older chemical doctrines, however, continued to prevail, in spite of the powerful and well-directed genius of Van Helmont, and the severe and pointed satire of GUY-PATIN.

The atomic theory of DES CARTES* produced a new modification of the chemical hypothesis. The philosophers of his school considered that the variety in the qualities of the different secretions depended on the peculiar conformation of the pores of the glands through which they passed; which pores permitted only those atoms to escape that corresponded in form and volume to their areas. Acids were particles that were adapted for penetrating into the vacuities of alkalies, &c. This turned the attention of physicians to mathematical and mechanical reasoning; and gave rise to notions respecting the functions of animal bodies but little less absurd than those of the Rosicrucians. Even BOYLE, although an opposer of zoötic chemistry, paid a tribute to the delusion of the age, in his reflections on the nature of specifics, and the influence of amulets on the human body, by means of their attraction of certain morbid particles.†

It was Sylvius de le BÖE who carried the application of chemistry to its fullest extent.‡ The human body, with him, was a chemical apparatus, where the heart is excited to action by the fermentation of the blood: from the aliments digested in the stomach there arise vapours distilled in the brain, which sends spirits to all the other organs of the body. Diseases depend on fermentations which corrupt the humours, producing either an acid or alkaline state of them. From the fluids in a state of effervescence, precipitations, dissolutions, and despumations, take place, similar to those in a barrel of wine. The physician's efforts are to be directed to lessen the violence of the fermentation by sedatives, or to excite it, when too languid, by stimulants; to precipitate feculencies by various remedies, of which the algaroth powder was the most efficacious; to neutralise acids by absorbents and alkalies; to destroy the corrosive quality of the lymph in syphilis; to dissipate the cold acid vapours of the pancreatic fluid in hypochondriasis, &c.

This hypothesis was received with almost universal approbation on the continent of Europe: it was patronized by

---

* *De Homine;* Latine edit. DONATUS, Lugd. Batav. 1664, p. 65 et seq.

† *Chemista Scepticus;* Londini, 1661.

‡ *Opera Omnia, Methodus Medendi;* Amstelodamiæ, 1679.

PASCAL,* ETMULLER, LENTILIUS, FRANCIS BAYLE,† MINOT;‡ VOLPI,§ VIEUSSENS,‖ HELVETIUS,¶ and even by RAMAZZINI. It was also adopted in England with some modifications. CHARLETON admitted the doctrine of fermentation of Van Helmont; MAYOW supposed that the inflammable particles of the air insinuated themselves into the blood, and produced a sort of vital inflammation with the sulphurous elements of that fluid; CROONE imagined that muscular motion arose from effervescence of the animal spirits with the sulphurous particles of the blood;** WILLIS framed a physiological hypothesis on the influence of the igneous vital spirits, of which there was a continual distillation going on in the brain: the blood he considered to ferment like wine, and that in fevers this was of a sulphurous nature; spasms arose from an explosion of salt and sulphur in the animal spirits; gout from a coagulation of the blood; and scurvy from a state of the blood similar to faded, musty wine; and that all the secretions were particular distillations.†† HARRIS,‡‡ FLOYER, LISTER,§§ and DUNCAN,‖‖ admitted either an acid or alkaline state of the humours as the cause of most diseases. The same notions influenced the reasonings of SYDENHAM.

The knowledge of the circulation of the blood, the doctrines of Des Cartes, and the philosophical experiments of GALILEO and BORELLI, produced at length a more extensive influence on medical reasonings; every phenomena was to be explained on mathematical principles: of this, the Treatise of MEAD on the bite of the Tarantula spider will afford a sufficient specimen. KEIL and JURIN were amongst its most strenuous supporters. This hypothesis, somewhat modified, formed the basis of the systems of BOERHAAVE and HOFFMANN. The influence of chemistry now rapidly declined over the whole of Europe; and, when the doctrines of George Ernest STAHL¶¶ became generally promulgated,

---

* *Traité des Fermens;* Paris, 1681.

† *De Corpore Animato;* Tolosæ, 1700.

‡ *De la Nature et Cause des Fièvres;* Paris, 1710.

§ *Spasmalogia;* Asti, 1710.

‖ *De Remotis et Proximis Mixti Principiis;* Lugdun. 1715.

¶ *Mémoires de l'Academie des Sciences;* An. 1716.

** *De Ratione Motus Musculorum;* Londini, 1664.

†† *De Fermentatione, et de Febribus;* 1668.

‡‡ *De Morbis Infantum;* 1689.

§§ *De Humoribus.*

‖‖ *Chymiæ Naturalis Specimen;* 1707.

¶¶ *Theoria Medica Vera;* Halle, 1708.

and almost universally adopted, this branch of science no longer shared the studies of the generality of physicians.

The philosophical opinions prevalent at that period, which would not admit the existence of intrinsic active force in matter, but attributed all its actions to the influence of an intelligent principle of an immaterial nature; joined with the doctrine of Des Cartes, adopted and extended by MALEBRANCHE, and the physiological hypothesis of Van Helmont, appear to have given rise to this important and remarkable revolution in the doctrines of the medical art.

The discoveries made in chemistry in the latter part of the last century, causing almost an entire change in the received principles of that science, and the greater facility with which analytical enquiries were conducted, led philosophers to examine anew the greater number of natural products with a degree of accuracy that had previously been unknown; an accuracy, in a great measure, dependent on the introduction of the use of re-agents in chemical experiments. Physiology soon shared these advantages: the labours of FOURCROY were the commencement of a rational and really useful application of chemistry to physiology and the practice of medicine; and, notwithstanding the important discoveries that have since been made by the numerous eminent philosophers who have devoted a share of their attention to this part of chemical science, his works on those subjects may yet be studied with advantage. Here we must terminate our observations. To trace the development of the progress of animal chemistry from that to the present period, and to investigate the extent of its utility, would demand the space of a large volume; besides which, we should despair of being able to add any thing of importance to the reflections adduced by BERZELIUS on this part of the subject, in his work expressly devoted to it.

To investigate the nature, and trace the different stages of the process by which alimentary substances, being received into the digestive organs, acquire the peculiar mode of combination and possess the properties evinced in the blood, is doubtless one of the most interesting and rational applications of chemistry to physiology: it has consequently engaged particular attention, both from philosophers particularly devoted to the study of that branch of science, and from those especially denominated *practical* physicians. Notwithstanding the many experiments and the reasonings that have been employed on this subject, both in a chemical and a general physiological point of view, we have yet much to learn respecting it. Our readers will, therefore, peruse with much gratification the account we are about to relate of some experiments of Dr. PROUT; the excellence of whose qualifications for its investigation they must have long since learned to appreciate. It is deduced from a Memoir published in the thirteenth volume of the Annals of Philosophy.

"Perhaps," observes Dr. Prout, "it may facilitate the perusal of these pages to premise, in general terms, the opinions which my observations have led me to form respecting the development and nature of the blood; the arrangement of the subject being chiefly founded upon that opinion. My notion is, then, that the blood begins to be formed or developed from the food, in all its parts, from the first moment of its entrance into the duodenum, or even, perhaps, from the first moment of digestion; and that it gradually becomes more and more perfect as it passes through the different stages to which it is subjected, till its formation be completed in the sanguiferous tubes, when it presents an aqueous solution of the principal textures and other parts of the animal body to which it belongs.

"The chief ingredients in the blood are *albumen*, *fibrin*, and the *colouring principle*, which may be supposed to represent the *common cellular texture*, the *muscular texture*, and the *nervous texture*,* respectively. These different principles are so nearly allied to one another in their chemical properties, that Berzelius has given them the general name of albuminous contents of the blood; a term which, for the sake of convenience, we shall adopt in the following enquiry.

"The principal distinct stages in the formation of blood, in all the more perfect animals, are *digestion*, *chymification*, *chylification*, and *sanguification*, usually so called: the first process being confined to the stomach, the second to the duodenum, the third to the lacteals, and the fourth to the blood-vessels.

"The properties of *chyme*,† *chyle*, and *blood*, the result

---

* "I by no means wish to be understood to assert that the red particles of the blood are destined to form the cerebral and nervous substances of animal bodies. I believe, however, that they are more intimately connected with the nervous function than any other ingredient of the blood, as I shall attempt to show hereafter.

† "I use the term *chyme* in a sense somewhat different from that commonly employed, by limiting it to that portion of the alimentary matter found in the duodenum, which has already, or is about to become albumen, and thus to constitute a part of the future blood.

of these processes, appear to run gradually and imperceptibly into one another; and hence, perhaps, they can hardly be considered as distinct and well-defined steps in the general process of sanguification. As, however, the vessels or organs in which they take place are perfectly distinct, it becomes a matter of convenience to consider the processes themselves as distinct also."

Dr. Prout first notices the phenomena, &c. of digestion in a rabbit, which had been kept without food for twelve hours, and was then fed upon a mixture of bran and oats. Two hours afterwards it was killed:—the stomach was moderately distended with a pulpy mass; the different articles of which it was composed could be barely recognized. The digestive process did not appear to have taken place equally throughout the mass, but seemed to be confined principally to where it was in contact with the stomach. Its smell was fatuous and disagreeable. On being wrapped up in a piece of linen, and subjected to moderate pressure, it yielded upwards of half a fluid ounce of an opaque reddish-brown fluid, which reddened litmus paper; but, on being dried or exposed to the air, the blue colour returned. The next day, however, the paper had assumed a red colour, which was permanent. It coagulated milk; and, moreover, seemed to possess the property of re-dissolving the curd, and converting it into a fluid, very similar to itself in appearance. It was not coagulated by heat or acids; and, in short, did not exhibit any evidence of an albuminous principle. On being evaporated to dryness, and burned, it yielded very copious traces of an alkaline phosphate and sulphate; also of various earthy salts,—as the sulphate, phosphate, and carbonate, of lime. The duodenum of the animal, at its commencement, contained chiefly a greenish-yellow glairy fluid, full of air-bubbles, with a small portion only of the insoluble parts of the food. This yielded decided evidence of the existence of a true chymous or albuminous principle: the quantity of which was found to increase to the distance of about six inches from the pylorus, after which it diminished; and at the distance of twenty-four inches from the pylorus, it was barely perceptible. When the animal was opened at a longer period after feeding, much stronger evidences of albuminous matter were found, not only in the duodenum, but nearly throughout the whole of the small intestines. The quantity, however, was generally very minute in the ileum; and where it enters the cœcum, no traces of this principle were discovered. A similar experiment was made on animals of different species; but the results did not present any other phenomena sufficiently interesting to merit being detailed in this place.

Dr. Prout next treats of the general and chemical properties of *chyme*. This fluid varied somewhat as it was formed either from vegetable or animal food. That from vegetable food (principally bread), found in the duodenum of a dog, was composed of a semi-fluid, opaque, yellowish-white part, containing another portion of a similar colour, but firmer consistence, mixed with it. Its specific gravity was 1.056. It showed no traces of a free acid, nor alkali; but coagulated milk completely, when assisted by a gentle heat. It was found to consist in proportions of 86.5 parts of water; 6.0 of what Dr. Prout terms the *gastric principle,* united with the alimentary matters, and apparently constituting the chyme, mixed with excrementitious matter; 1.6 of biliary principle; 5.0 of vegetable gluten; 0.7 of saline matters; and 0.2 of insoluble residuum. That from the duodenum of a dog fed on animal food was more thick and viscid than that from vegetable food, and its colour was more inclining to red. Its specific gravity was 1.022. It showed no traces of a free acid or alkali; nor did it coagulate milk, even when assisted by the most favourable circumstances. It consisted of 80.0 parts of water; 15.8 of gastric principle; 1.3 of albuminous matter, partly consisting of fibrin; 1·7 biliary principle; 0.7 saline matter; 0.5 insoluble residuum.

The properties of *chyle* are next considered, in the three different stages of its progress towards the sanguiferous system. Owing to the minuteness of the lacteal vessels, its qualities in the first stage are but imperfectly known. In the examination of it made in the horse, by Emmert and Reuss, it was found to be opaque, and white like milk; differing from chyle taken from the thoracic duct, in being more white and opaque, in undergoing spontaneous coagulation much more slowly and imperfectly, and in not assuming a reddish colour on exposure to the air. Chyle from the sublumbar branches of horses, examined by the before-mentioned physiologists, and also by Vauquelin, is represented as possessing properties more imperfect and ill-defined than those of chyle taken from the thoracic duct: it was found to undergo spontaneous coagulation much more imperfectly. Its colour was white, with minute yellow globules swimming in it. But, in a few hours, there was observed in it a little reddish mass swimming in a yellowish fluid, which after some days disappeared, and assumed the form of a sediment at the bottom of a vessel. Chyle taken from the thoracic duct of an animal killed a few hours after having taken food, is in a perfectly fluid state; its colour is nearly white; its taste faintly saline and sweetish. In a period of time somewhat different in different instances, but generally in a few minutes, it begins to assume a gelatinous appearance, and to undergo coagulation; the colour also, if it have been exposed to the air, changes to a faint red, or pink. These observations are taken from Emmert, Reuss, and Vauquelin. The descriptions of this fluid by Dr. Marcet and Dr. Prout do not materially differ from the above. On chemical analysis, Dr. Prout found the chyle taken from the thoracic duct of a dog fed on vegetable food, to consist of 93.6 parts of water; 0.6 fibrin; 4.6 incipient albumen?; 0.4 albumen, with a little red colouring matter; 0.8 saline matter, and some traces of sugar of milk? and of oily matter. The respective proportions in a dog fed on animal food were—89.2; 0.8; 4.7; 4.6; and 0.7. The saline matters consisted chiefly of the alkaline muriates, with traces of a sulphate, and perhaps of a lactate: but of this the author is not certain. Here the Memoir of Dr. Prout terminates: we, therefore, defer the remarks we may have to offer respecting it, until we are enabled to lay before our readers the full development of the views and opinions of this learned and intelligent physician, to whom we are already indebted for so much valuable information relative to this branch of science.

Although the experiments and observations of Dr. Wilson Philip on digestion were published in the first edition of his physiological work, and consequently should not be included in an history extending backwards only to the period within which we profess to confine our views, yet, as they have never been detailed in this Journal, and will form an highly-valuable addition to the observations of Dr. Prout, we shall adduce them on the present occasion. Many of them are strictly physiological, in the ordinary application of that term; but, as several of them especially appertain to animal chemistry, and they are so indivisibly connected, we trust that we shall not be censured for noticing them under the head of Chemistry.

"The first thing which strikes the eye on inspecting the stomachs of rabbits which have lately eaten," observes Dr. Philip, "is, that the new is never mixed with the old food. The former is always found in the centre, surrounded on all sides by the old food, except that on the upper part, between the new food and the smaller curvature of the stomach, there is sometimes little or no old food. If, as we ascertained by more than twenty trials, the old and the new food are of different kinds, and the animal is killed after taking the latter (unless a great length of time was elapsed after taking it), the line of separation is perfectly evident, so that all the old may be removed without disturbing the new food."—"All around, the nearer the food lies to the surface of the stomach, the more it is digested. This is true even with regard to the food in the small curvature, compared with that nearer the centre; and the food which touches the surface of the stomach is more digested than any other found in the same part of the stomach. But, unless the animal has not eaten for a great length of time, the food in contact with the surface of the stomach is in very different stages of digestion in different parts of this organ. It is least digested in the small curvature, more in the large end, and still more in the middle of the great curvature.

"These observations apply to the cardiac portion of the stomach. Sir Everard Home, in his work on Comparative Anatomy, has shown that the stomach is divided into two portions, the cardiac and pyloric, in such a way, that the length of the former is to that of the latter nearly as two to one. The line of division may generally be seen in some animals after death." Such an appearance was observed by Dr. Philip; and he continues to observe, "that these two portions form an angle with each other, which is well expressed by the plates in Sir Everard Home's work. The food in the pyloric portion of the stomach of the rabbit, is always found in a state very different from that just described. It is more equally digested; the central parts differing less in this respect from those which lie next to the surface of the stomach: it is evident, however, that all the change effected in the stomach is not completed when the food enters this portion of it, because we find it the more digested the nearer it approaches to the pylorus."—"One of the most remarkable differences between the state of the food in the cardiac and pyloric portions of the stomach, is, that in the latter it is comparatively dry; in the former, mixed with a large proportion of fluid, particularly when digestion is pretty far advanced, and time has consequently been given for a considerable secretion from the stomach.

"It appears that, in proportion as the food is digested, it is moved along the great curvature, where the change in it is rendered more perfect, to the pyloric portion. Thus, the layer of food lying next the surface of the stomach is first digested. In proportion as this undergoes the proper change, it is moved on by the muscular action of the stomach; and that next in turn succeeds to undergo the same change. As the gastric juice pervades the contents of the stomach, though apparently in no other way than by simple juxtaposition; the change in each part, which in its turn comes in contact with the stomach, is far advanced before it is in actual contact with it, and, consequently, is soon after this in a proper state to be moved on towards the pyloric end. Thus a continual motion is going on,—that part of the food which lies next the surface of the stomach passing towards the pylorus, and the more central parts approaching the surface.

"It is in the great end of the stomach where digestion goes on so rapidly, that Mr. Hunter found the stomach itself dissolved; and, by the most satisfactory arguments, showed that this is the effect of the gastric juice after death."*

These observations of Dr. Philip are highly valuable; for, at the same time that they add to the evidence we previously possessed of the agency of the gastric fluid in digestion, they show that that process is not solely dependent on chemical agency, but that it is effected by means of the more general laws that influence the functions of the animal economy: in other words, that experiments with the gastric fluid out of the body, will afford but little information on this subject; and that the stomach is not merely a vessel in which the chemical changes in the aliment are affected, but also a *vital organ* performing an active and important office in that process. But we need not dwell further on this part of the subject on the present occasion, having noticed it in a particular manner in our analysis of the thesis of Dr. LALLEMAND.†

Calculous concretions in the urinary organs is one of the subjects to which chemistry has been applied with the most decisive beneficial influence on the practice of medicine. Indeed, the therapeutics for the maladies thence arising are founded on the principles of that branch of science: although it is obvious that we have hitherto only applied them to remedy the effects of a primary disease; for these concretions themselves appear to arise, in most instances, from either general or topical derangement of the animal economy, the consideration of which has, perhaps, been too much neglected since the means of remedying its ill effects have been more precisely ascertained.

---

* *An Experimental Enquiry into the Laws of the Vital Functions,* &c. &c.; by A. P. Wilson Philip, M.D. F.R.S.E. &c. Second edition, p. 140 et seq. London, 1818.

† *London Medical and Physical Journal,* vol. xl. p. 531 et seq.

The work of Dr. MAGENDIE* on this subject is of considerable value, although it bears marks of hasty composition, and want of logical precision in the deduction of general conclusions from the facts to which it relates.

The observations of Dr. Magendie in this work relate to those concretions alone which appear in the form of gravel, and which are composed of *uric acid*, combined with a small proportion of animal matter. The chemical composition of this acid is found to be in the proportions of 39.16 azote; 33.61 carbon; 18.89 oxygen; and 8.34 hydrogen. Azote, then, constitutes one of the chief of its elements. This induced the author to examine the urine of herbivorous and carnivorous animals, with the view to ascertain the influence of aliments in the production of uric acid. He found that the urine of the former class always contains it, whilst that of the latter is perfectly free from its presence. This led to some enquiries, in order to determine whether the use of vegetable food alone, in carnivorous animals, would prevent the formation of the acid; and, from some experiments made on dogs by feeding them with sugar, he is disposed to decide this question in the affirmative: the propriety of which conclusion is very doubtful, when the diseased state produced by this diet, in the animal whose case is detailed, is considered; particularly, too, when we find that BERZELIUS, in his work on Animal Chemistry,† states that the blood of herbivorous animals contains a greater proportion of azote than that of man. M. Magendie observes, that the azote contained in the food is principally expended on the muscles, and that it is when these parts are not sufficiently exercised, or that azoted food is taken in larger quantities than is necessary for their nutrition, that it appears in an inordinate proportion in the urine, and in the form of uric acid. The habits of brute herbivorous animals may, therefore, according to the statements and principles of that physiologist, be adduced to explain why azote does not appear in their urine in the form of uric acid; but an inference cannot thence be derived to show that the use of vegetable food in animals naturally carnivorous should in them prevent the production of that

---

* *Recherches Physiologiques et Medicales sur les Causes, les Symptomes, et le Traitement de la Gravelle;* par F. Magendie, M.D. &c. Paris, 1818.—See *London Medical and Physical Journal*, vol. xl. p. 338 et seq.

† *A View of the Progress and present State of Animal Chemistry;* by J. Jacob Berzelius; translated from the Swedish. London, 1813.

substance. If the blood of herbivorous animals contain a greater proportion of azote than that of man, and their muscles and other parts are equally formed in part of that principle, there must be some outlet for the evacuation of this matter from the body, when separated from its organs in the process of mutation, (which would appear to be through the medium of the kidneys.) Admitting that the statements of M. Magendie are correct, it would then appear that the presence of uric acid in the urine of one class of animals; and the absence of it in another, whose solid parts contain it in an equal proportion, and whose blood contains more,—can only be explained by attributing these circumstances to a precise and absolute difference in the functions of particular organs in those animals: unless we admit a passage from the stomach or intestines to the kidneys, distinct from that of the general circulation; and that the quantity of azote taken with the aliment which is greater than what is necessary for the support of the body, is immediately carried off by means of that communication. The former of these propositions has not been satisfactorily established; and, to admit the latter, would be to attribute an elective function to those parts that is merely imaginary.

Another question then arises—Will animals, accustomed to take a large quantity of azote as food, preserve their health by the use of a diet containing only a very small proportion of that substance, or, as M. Magendie proposes in some cases, to the absolute want of it? The facts adduced by him to show the correctness of his principles, and the good effects of the mode of treatment he deduces from them, would authorize a conclusion in the negative; as far as such a number of facts can with propriety be applied. The dog died apparently in consequence of the diet to which he was confined; and the general health of the patients who continued the use of aliments containing but a small proportion of azote, beyond a certain length of time, became considerably deteriorated. We should have before observed, that uric acid requiring about fifteen hundred times its weight of urine for its solution, when it exists in that fluid in a greater proportion, it necessarily becomes precipitated in a solid form: this constitutes the concretions termed gravel.

With respect to the patients to whom we have just alluded; the existence of gravel in the urine, to which they had previously been subject, disappeared under the use of the non-azoted diet; but we should be disposed, for the reasons we have already mentioned, to attribute this to physiological, not to chemical, causes. They were persons who had indulged to a great extent in a luxurious diet; and, in addition to this, some of them had led an indolent life; and, therefore, the measures which would remedy these causes of derangement of the general animal economy, would also probably obviate the production of gravel as one of their effects.

The above remarks are offered with much diffidence, and principally with the intention of preventing the undue influence which a specious hypothesis might have on some of our readers; by furnishing them with some hints for reflection, which will show them that it should be admitted with extreme caution.

---

